# COVID-19: Look to the Future, Learn from the Past

**DOI:** 10.3390/v12111226

**Published:** 2020-10-29

**Authors:** Zhangkai J. Cheng, Hui-Qi Qu, Lifeng Tian, Zhifeng Duan, Hakon Hakonarson

**Affiliations:** 1 Institute of Medical Physics, School of Physics, University of Sydney, Sydney, NSW 2006, Australia; 2 Center for Applied Genomics, The Children’s Hospital of Philadelphia, Philadelphia, PA 19104, USA; quh@email.chop.edu (H.-Q.Q.); tianl@email.chop.edu (L.T.); bravewindheart@163.com (Z.D.); 3 Department of Pediatrics, The Perelman School of Medicine, University of Pennsylvania, Philadelphia, PA 19104, USA; 4 Division of Human Genetics, Children’s Hospital of Philadelphia, Philadelphia, PA 19104, USA; 5 Division of Pulmonary Medicine, Children’s Hospital of Philadelphia, Philadelphia, PA 19104, USA

**Keywords:** COVID-19, coronavirus, epidemiology, SARS-CoV-2

## Abstract

There is a current pandemic of a new type of coronavirus, the severe acute respiratory syndrome coronavirus 2 (SARS-CoV-2). The number of confirmed infected cases has been rapidly increasing. This paper analyzes the characteristics of SARS-CoV-2 in comparison with Severe Acute Respiratory Syndrome Coronavirus (SARS-CoV), Middle East Respiratory Syndrome Coronavirus (MERS-CoV) and influenza. COVID-19 is similar to the diseases caused by SARS-CoV and MERS-CoV virologically and etiologically, but closer to influenza in epidemiology and virulence. The comparison provides a new perspective for the future of the disease control, and offers some ideas in the prevention and control management strategy. The large number of infectious people from the origin, and the highly infectious and occult nature have been two major problems, making the virus difficult to eradicate. We thus need to contemplate the possibility of long-term co-existence with COVID-19.

## 1. Introduction

**Figure 1:**
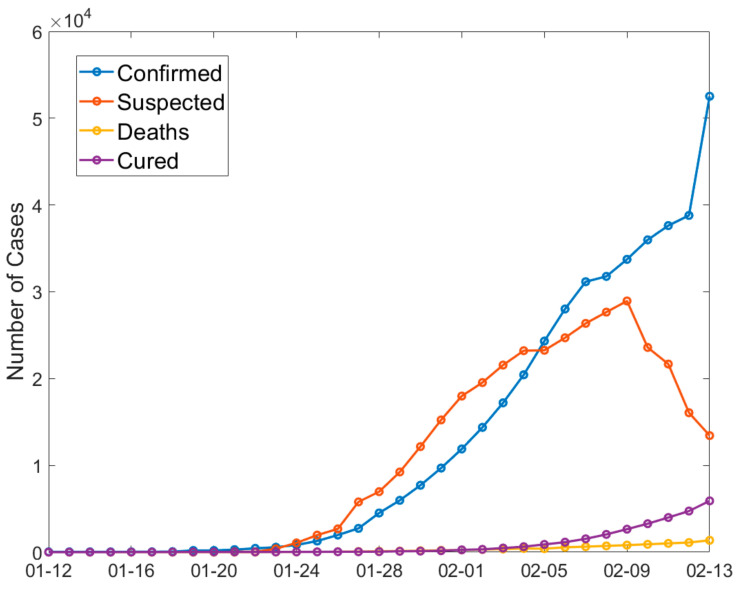
Number of confirmed cases, suspected cases, deaths and cure over time in early days in China.

In December 2019, a cluster of patients presenting with viral pneumonia were reported in Wuhan, China. This has been identified to be Coronavirus Disease 2019 (COVID-19), caused by a new type of coronavirus, the severe acute respiratory syndrome coronavirus 2 (SARS-CoV-2). The coronavirus quickly spread across people living in or having visited Wuhan, and human-to-human transmission was soon confirmed [1]. Figure 1 shows the outburst of COVID-19 in early days at the beginning in China, based on data taken from the past daily statements of National Health Commission, China [2]. According to Wu and McGoogan [3], 81% Chinese patients with COVID-19 had mild symptoms, 14% had severe symptoms, and 5% were critically ill. Since then, the virus has been spreading rapidly and caused a pandemic across the world. As of 20 October 2020, it has been diagnosed in >40.3 million people globally, causing >1.1 million deaths. We studied the characteristics of COVID-19 in comparison with Severe Acute Respiratory Syndrome Coronavirus (SARS-CoV), Middle East Respiratory Syndrome Coronavirus (MERS-CoV) and influenza, in terms of virology, etiology, epidemiology and virulence. Prevention and control strategies are analyzed and discussed in the following text.

## 2. Comparison to SARS-CoV and MERS-CoV

After Severe Acute Respiratory Syndrome Coronavirus (SARS-CoV) and Middle East Respiratory Syndrome Coronavirus (MERS-CoV), COVID-19 is the seventh known member of the family of coronaviruses that are pathogenic to humans. The rest of the seven members (229E, NL63, OC43 and HKU1) have been associated with mild clinical symptoms [4]. SARS-CoV emerged in China in November 2002, and resulted in more than 8000 human infections and 774 deaths in 37 countries [5,6]. MERS-CoV was first detected in Saudi Arabia in 2012 and was responsible for 2494 confirmed cases of infection and 858 fatalities, including 38 deaths following a single imported case into South Korea [7,8,9]. SARS-CoV-2 (GenBank accession MN908947) is a beta-coronavirus (β-CoV) of group 2B, and has been found to have the highest similarity (89%) to a SARS-related member of the Sarbecoviruses (GenBank accession MG772933), a subgenus within the β-CoV genus [10,11].

Early patient data from Wuhan in China has shown similarities of clinical features between COVID-19 and previous β-CoV infections. Similar to SARS-CoV and MERS-CoV infections, patients have exhibited symptoms of viral pneumonia including fever, cough, difficulty breathing and bilateral ground-glass opacities on chest CT scans [12]. However, the virulence of COVID-19 is far less than that of SARS, with Case Fatality Rate (CFR) of 0.72% according to the Centre for Evidence-Based Medicine (CEBM), Update 9 April (https://www.cebm.net/covid-19/global-covid-19-case-fatality-rates/), whereas the case fatality rate is around 10% for SARS [13] and 37% for MERS [14].

Similar to the 2003 SARS outbreak in Guangzhou in China, the initial COVID-19 outbreak was tentatively associated with a wet markets [1,15], suggesting that the virus originated from wildlife animals sold in the market [16]. However, a later *The Lancet* report claimed 13 of the 41 first reported patients with no epidemiological link to the marketplace [12]. The virus may have spread silently in Wuhan before the discovery of the cases, during its incubation period [17,18].

All three aforementioned viruses (SARS-CoV, MERS-CoV and SARS-COV-2) are believed to have natural hosts in bats [19,20]. It is most likely that they are able to invade the human world only after a genetic mutation in an intermediate host animal. The intermediate hosts for SARS and MERS are civets [21] and camels [22], respectively. Determining the intermediate host is crucial to our future efforts to prevent the return of coronavirus diseases. Unfortunately, scientists have only been able to detect the virus in the market environment, but not in time to collect wildlife samples before the market was shut down [16]. There had been various candidates proposed, such as Malayan Pangolins [23,24], but most of them have been discredited [25,26]. In the meantime, the scientific community should be vigilant about the possibility of return of another coronavirus in another one or two decades [27].

During the previous outbreaks of SARS and MERS, human to human transmission was efficient through droplets, contact and fomites, suggesting that the transmission mode of the COVID-19 can be similar [28].

In terms of transmissibility, we previously estimated the reproduction number R_0_ provided by eight different research groups, and found most of the R_0_ value to lie in the range between 2 and 3 [1]. Back in January, Wu et al. published a comprehensive model for predicting the future spread of COVID-19 in The Lancet and estimated R_0_ to be around 2.68 [29]. Most recently, based on 16 published estimates, the estimate R_0_ (95% CI) by CEBM is 2.65 (1.97, 3.09) (14 April 2020, https://www.cebm.net/covid-19/when-will-it-be-over-an-introduction-to-viral-reproduction-numbers-r0-and-re/). This R_0_ estimation is similar to or slightly less than that of SARS (around 3) [30] and larger than MERS, which is less than one [31]. To explain its transmissibility, we investigated RNA sequencing transcriptome profiling of human samples of airway and oral mucosa, and the expression pattern of the SARS-CoV-2 receptor ACE2 and the viral priming protease, TMPRSS2. We observed that ACE2 had medium levels of expression in both small airway epithelium and masticatory mucosa, and high levels of expression in nasal epithelium, and TMPRSS2 was highly expressed in small airway epithelium and nasal epithelium. These findings provided the molecular basis that the nasal mucosa is the most susceptible locus in the respiratory tract for SARS-CoV-2 infection [32]. In addition, the lack of effective immune responses to eliminate the infection also contribute to the high transmissibility, e.g., IgA responses to clear respiratory tract infection may be harmful in COVID-19 as shown by our recent study [33].

While SARS-CoV-2 is similar in transmissibility compared to SARS, it is much less virulent than SARS. Why, then, has the number of confirmed cases of the disease greatly surpassed SARS and continued to increase as a pandemic? There has been a variety of potential epidemiological reasons:

(1) The occult nature of SARS-COV-2. SARS-COV-2 is not as virulent as SARS, and produces a large number of patients asymptomatic or with mild symptoms [34]. According to the study on passengers on board the Diamond Princess Cruises’ ship, 50.5% of infected individuals were asymptomatic [35]. Similarly, ~60% infected sailors on the U.S. aircraft carrier Theodore Roosevelt were asymptomatic. Compared with SARS patients, asymptomatic patients with COVID-19 are likely not to go to hospitals for testing for COVID-19. In US, people with mild illness are not suggested to do testing. Even if they do the testing, false-negative has been an issue to identify the cases [36]. At the same time, the incubation period of COVID-19 is longer than SARS, generally between 2–16 days (the 5th and 95th percentiles) [28,37]. Furthermore, a risk of pre-symptomatic transmission was also suggested by Rothe and colleagues for the symptomatic patients [38], although this finding has been met with criticisms [39]. Because of these reasons, a large number of SARS-COV-2 infected people has not been identified in time and quarantined, which enabled the wide spread of transmission. SARS on the other hand, exhibits serious illness, resulting in a lower chance of misreporting.

(2) Detection, publishing, and warning measures lagged far behind in the early stages of the outbreak, which gave time for the virus to spread. As a result, before the large-scale national prevention and control measures in China were implemented, especially before the closure of Wuhan city, a large number of patients had been accumulated in the Wuhan area. The Lancet paper on 31 January estimates that as of 25 January, there were more than 75,000 patients in Wuhan alone [29]. After the Wuhan city was closed down, a few governments evacuated their citizens and performed thorough medical examination for each individual brought back. The authors searched available information on Wuhan evacuation released by governments of different countries on the public domain. Table 1 lists the citizens evacuated by each country in the last January and early February. Evacuation can be thought of as a random sampling of the population of the Wuhan area (11 million in total), and the prevalence rate is close to what the Lancet paper suggested. In other words, the infection rate in the Wuhan area may have reached one percent, or 110,000 people up to early February 2020. Those undiagnosed patients exacerbated the spread of the transmission of COVID-19.

(3) Timing and Chinese culture. Both the COVID-19 outbreak in Wuhan and the SARS 2003 outbreak in Guangzhou happened in megacities in China (Guangzhou had a population of 9,942,022 in 2000). Around January each year, a huge amount of population would leave major cities and travel back to their respective hometown to celebrate Chinese New Year, and come back around February. The total number of Chinese passengers were estimated to be 2.91 billion Person-Times in 2016. This is known as the Chunyun period, or Spring Festival travel season. The COVID-19 outbreak happened in late December and early January, albeit the low number of confirmed cases due to lack of detection. Before Wuhan closed its city, 5 million people had already left Wuhan during the Chunyun period [50], which hindered any containment effort. At the same time, socio-economic globalization promoted the spread the COVID-19 across the world [51]. SARS on the other hand, although detected in 2002, the real outbreak did not happen until March 2003. The Chunyun period had by then ended, and the containment efforts were thus more effective.

## 3. Prevention and Control

It was learned from the SARS outbreak—which started as animal-to-human transmission during the first phase of the epidemic—that all game meat trades should be optimally regulated to terminate this portal of transmission, to prevent the outbreak of another coronavirus pandemic [52].

For COVID-19, global experts and governments have been actively tracking the spread and virulence of the virus, and to provide advices to individuals on measures to protect health and prevent the spread of new outbreak. In response to the spreading virus, isolation strategy has been used. Many Chinese cities including Wuhan had been put on quarantine since 23 January 2020, and many countries have closed their boarder to people travelled internationally [1].

Since the early stage of COVID-19 pandemic, to systematically organize countries to combat COVID-19, WHO has developed a “global strategic preparedness and response plan” to support all countries to prepare for and respond to the outbreak. The goal is to stop further transmission of COVID-19 within China and to other countries, and to mitigate the impact of the outbreak in all countries. They have outlined a list of public health measures, including rapid identification, diagnosis and management of the cases, identification and follow up of the contacts, infection prevention and control in healthcare settings, implementation of health measures for travelers, awareness raising in the population, risk communication [28]. With the development of pandemic, stay-at-home orders have been issued in many countries, which have contributed significantly to contain the speed of transmission of COVID-19, thus to avoid straining medical resources.

The understanding of the spread and features of the virus is still updating rapidly, but that does not prevent us from starting to think further about the problem of the future pandemic of new coronavirus and preparing plans for it, through international cooperation.

### 3.1. A New Perspective

Effectively and comprehensively isolating patients and their close contacts, cutting off their path to continuing transmission, and waiting for the incubation period to pass, was a very effective measure to eliminate SARS in a short period of time. This strategy may work on several premises:

1. The total number of patients was small. Therefore, the administration was able to find and quarantine the close contacts of each patient;
2. Once becomes onset, the symptoms were obvious, critical and typical. Patients were quickly identified and treated;
3. There was no transmission capability during incubation period.

COVID-19 however, does not satisfy these preconditions. Having a huge number of patients, as well as a significant proportion of asymptomatic patients, and highly infectious occult infection, has greatly increased the difficulty of prevention and control. We will not be able to identify every patient that exhibits mild symptoms for quarantine purpose, nor to put all their close contacts under isolation and observation.

On the other hand, COVID-19 shares many similar characteristics of influenza. Here are the features of the seasonal flu, which kills 646,000 people worldwide each year [53]: it has low virulence, with case fatality rate of about 0.1% [54]; its reproduction number R0 is around 1.3, which is less than SARS [55]; the number of cases is much larger however, while about 10% of unvaccinated adults are infected each year; although influenza virus usually has a short incubation period of between 1–4 days, transmission can occur within the incubation period [56]; flu symptoms are less distinct, with fever, cough, sore throat and other symptoms that are difficult to distinguish from other respiratory diseases prevalent in autumn and winter. Table 2 below compares different characteristics of the three coronaviruses, as well as influenza.

Although COVID-19 is more similar to SARS in virology and etiology, its epidemiological impacts are less like SARS but more like a “mega flu”. This could mean we may take historical experiences from the fight against influenza cautiously. However, we need to keep in mind not to downplay the complexity and severity of COVID-19. The majority of human population have some extent of immunity to influenza virus by natural exposure and/or vaccination. In addition, there are antivirals clinically approved for threating influenza. On the other hand, there is no effective antivirals for threating COVID-19. According to the most recent clinical trial by the WHO Solidarity trial consortium, Remdesivir, Hydroxychloroquine, Lopinavir and Interferon regimens showed little or no effect on hospitalized COVID-19 [57].

Closing schools during flu outbreaks has been shown to be an effective measure [58,59]. However, when to shut down and how long to shut down are still indicators that need to be carefully considered [60]. Two studies from Australia and Japan showed that monitoring body temperature at airports was not effective in reducing the spread of the disease during the 2009 H1N1 swine flu pandemic [61,62]. A similar question to consider is how much effort we need to put into monitoring passengers’ temperatures in this outbreak, given that the incubation period for COVID-19 is much longer than for influenza and the large proportion of asymptomatic patients.

### 3.2. Vaccines

Vaccines are the first choice against a pandemic of this magnitude. According to U.S Centers for Disease Control and Prevention (CDC), the effectiveness of the flu vaccine varies from year to year, but overall it reduces the risk by 40 to 50 percent [63]. Vaccines need to go through many phases of development. The lead time to produce a vaccine lot ranges from several months (e.g., influenza vaccine) to three years [64]. Thanks to the rapid publication of the COVID-19 genetic sequence, a number of biopharmaceutical firms and academic research facilities around the world have launched programs and are racing to find an effective vaccine. Many are aiming to break the vaccine development record time [65]. As of 16 October, there were 11 vaccines under phase 3 trial [66], each falls into one of three categories [67]. The first category is inactivated vaccine. It is the most traditional and sophisticated method of preparing a vaccine by growing the virus and then injecting it with a “killed” virus. Second, non-replicating viral vectors. It is the non-toxic adenovirus as a carrier, into the protein gene, and then injected into the body. The third category is RNA-based vaccine. Since vaccine development is not always effective, and the effectiveness will ultimately depend on trial data, the simultaneous development and implementation of multiple routes is expected to eventually increase the success rate of the vaccine.

### 3.3. Hierarchical Management

“Hierarchical management” of influenza takes into account the fact that when influenza is endemic, outbreaks in different parts of the world vary from region to region, and the severity of illness varies from patient to patient. Cities with clusters of cases may take more drastic measures, from canceling public gatherings to closing schools and workplaces, while areas with fewer cases can adapt to local conditions. Patients with serious illness are given intensive treatment in hospitals, while most patients with mild symptoms can seek treatment in small clinics or even wait for the disease to self-heal.

This strategy can certainly be translated to fight against COVID-19. During a full-scale pandemic, especially in underdeveloped regions as seen in the city of Wuhan, there will not be enough medical resources to attend every patient [68]. Medical needs should be given out to those with more severe symptoms or with high risk of fatal complications. The treatment of the large number of patients with mild symptoms can be home-based. What Wuhan was doing was to create makeshift hospitals using empty facilities such as sports stadium and exhibition halls [69], although how to avoid cross secondary infection has been a challenge. Patients with less severe symptoms in Wuhan were gathered and assigned a bed and facemasks in the makeshift hospital, and less medical resource was needed for each patient in this way.

**Figure 2:**
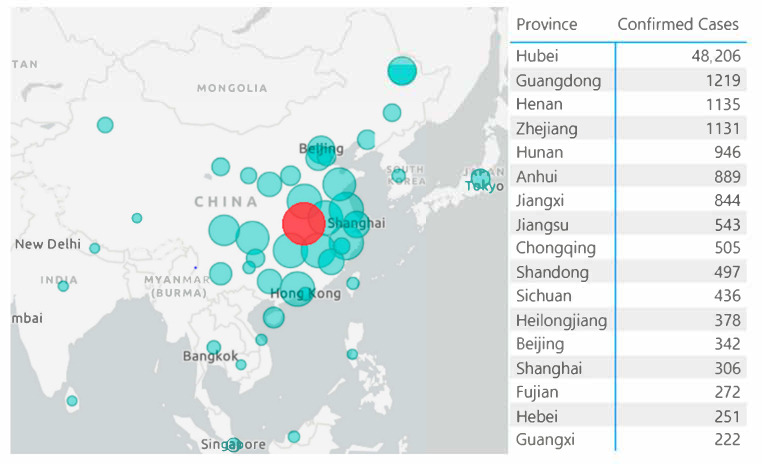
Bubble map visualization of the disease distribution around China, with data taken from [70], obtained on 13 February 2020. Created using Power BI Desktop.

What China has done was very different from the strategy taken by other countries. Looking back the management in China, the distribution of the disease was centered in Wuhan city, Hubei province (see Figure 2 to help understanding the strategy taken by China) and its surrounding cities, while cases in other cities were mainly imported. It has taken a long time and very harsh prevention and control measures until the outbreak in Wuhan was effectively controlled. In other areas however, the situation has been largely controlled in a few weeks, due to the isolation strategy in China after 23 January 2020. Within 14 days after the closure of Wuhan in Hubei Province, potential patients in other cities would have already developed clinical symptoms, with high probability of being quarantined and treated. At that point, it may be more important to consider putting the society back into normal operation and function as soon as possible, to avoid further damage to people’s life as well as the economy. We might consider exercising specific disease control and management in different regions across the world, instead of a “one-size-fits-all” approach.

### 3.4. A Long Winter

Governments around the world are doing their best in combatting and hopefully eradicating COVID-19, but we still need to be prepared for a long-term epidemic. The development of COVID-19 is closer to a “mega flu” that is more difficult to eradicate. That brings a possibility that this virus, like the flu, will persist in the human world for a long time to come, until the success of effective vaccine, or herd immunity achieved until large proportion of human population has been infected [71]. Currently, humans are still at a loss for influenza viruses that are less transmissible than COVID-19, and given the early accumulation of cases in the Wuhan area, it may be necessary to start embracing the possibility of long-term co-existence with COVID-19. In the meantime, the emergence of a more transmissible form of SARS-CoV-2 with the mutation Spike D614G, which began spreading in Europe in early February, has been reported [72].

Many of the methods of prevention and control in China have been similar to wartime measures, such as rapid construction of new hospitals, extensive restrictions on the movement of people, delaying the start of school, high levels of martial law in communities, banning outsiders, and quarantining the residents of a building if a patient was found. Putting other considerations such as civil liberties aside, the extreme measures have achieved success in dealing with the pandemic, and China is becoming the first major economy to recover from the pandemic. However, the same policies and procedures cannot easily be replicated in western democratic systems. Other government should focus on developments towards more universal, milder measures for the control of new burst of COVID-19, such as public education, social distancing, rapid response mechanisms, tiered management, and regular disinfection of public appliances, etc.

In our opinion, the media around the world is doing an excellent job in covering all aspects of COVID-19, and raising public awareness, however, this exerts enormous pressure on governments to make over-drastic policies [73]. Instead of proper alarm, the media portrait of a “deadly/killer virus” may cause panic and can harm efforts to implement a cost-effective and safe infection control strategy [74]. It may be time for the media and education to start adjusting their tone to reduce unnecessary panic. Instead, the emphasis should move towards the popularization of public health, such as wearing masks when sick, washing hands frequently, coughing and sneezing etiquette, etc.

After SARS was contained through extraordinary efforts, much of the research and drug development related to SARS stopped because of lack of funding and market prospects. A simple bibliometric analysis from PubMed databases, using “SARS” as a search term, reveals around 800 articles in 2003, which dropped to 27 in 2019. Given that the new coronavirus invades the human body in a similar way to SARS, if many of the previous studies had persisted, we might have been better prepared scientifically and medically for the new coronavirus. Perhaps we should learn from this lesson and rethink whether our entire scientific system is paying enough attention to re-emerging coronavirus diseases.

## 4. Conclusions

Through consolidating foreign citizen evacuation data, the early prevalence rate of COVID-19 in Wuhan population is found to be around one percent, or 110,000 infected people, which suggests that a large number of infected people have been neglected because of asymptomatic or mildly ill. It also explains the outburst of COVID-19 across the world after it has been contained in China. A large number of infected people in the origin city, plus the highly infectious and occult nature of COVID-19, made COVID-19 difficult to eradicate. The virulence and transmissibility of the new virus are somehow close to that of the influenza, and it would not be entirely unacceptable to tolerate some persistence. We need to contemplate the possibility of long-term co-existence with COVID-19, rather than allowing overly drastic measures to affect our normal lives and the normal social-economic activities of a country. It is worth emphasizing that the possibility of long-term co-existence with COVID-19 may not be as scary as it sounds. A recent study showed that the median infection fatality rate was 0.27% [75] for SARS-CoV-2, which is close to the approximate fatality rate of the flu of 0.1%. It is possible that over time with more general immunity in the population, the ultimate severity and mortality between the two viruses will be similar. While having a new pandemic flu-like pneumonia that occasionally flares-up is not a future anyone wants to be in, and indeed the pandemic so far has already caused massive damage to nations worldwide, it should not be a cause for social collapse.

These conclusions are based on our current knowledge of the new coronavirus and COVID-19, which may change, as may the virus and the disease itself. We need to be very cautious in the face of an enemy we have never seen before.

## Figures and Tables

**Table 1:** Citizens evacuated from Wuhan by each country.

Country	Evacuated	Confirmed Cases	Prevalence	Converted to Infected Population in Wuhan	Source
Japan	566	9	1.4%	156,000	[40]
South Korea	368	1	0.3%	30,000	[41,42,43]
US	195	0	0	0	[44]
Germany	124	2	1.6%	177,000	[45]
Singapore	92	1	1.1%	119,000	[46,47,48]
Italy	56	1	1.8%	196,000	[49]
Total	1401	14	1.0%	110,000	NA

**Table 2:** Comparison of four diseases.

Characteristics	SARS-CoV-2	SARS-CoV	MERS-CoV	Influenza
R_0_	2.68	3	<1	1.3
Virulence	Low	High	High	Low
Case Fatality Rate	2%	10%	37%	0.1%
Natural Hosts	Bat	Bat	Bat	Animals, humans
Intermediate Hosts	Unknown	Civets	Camels	NA
Origin of Outbreak	Wuhan, China	Guangzhou, China	Saudi Arabia	NA
Incubation Period	2–16 days	2–7 days	4–8 days	1–4 days

## References

[1] Cheng Z.J., Shan J. (2020). 2019 Novel Coronavirus: Where We are and What We Know. Infection.

[2] NHC (2020). Outbreak Notification. http://www.nhc.gov.cn/xcs/yqtb/list_gzbd.shtml.

[3] Wu Z., McGoogan J.M. (2020). Characteristics of and Important Lessons From the Coronavirus Disease 2019 (COVID-19) Outbreak in China: Summary of a Report of 72,314 Cases From the Chinese Center for Disease Control and Prevention. JAMA.

[4] Su S., Wong G., Shi W., Liu J., Lai A.C.K., Zhou J., Liu W., Bi Y., Gao G.F. (2016). Epidemiology, Genetic Recombination, and Pathogenesis of Coronaviruses. Trends Microbiol..

[5] Zhong N.S., Zheng B.J., Li Y.M., Poon L.L.M., Xie Z.H., Chan K.H., Li P.H., Tan S.Y., Chang Q., Xie J.P. (2003). Epidemiology and cause of severe acute respiratory syndrome (SARS) in Guangdong, People’s Republic of China, in February, 2003. Lancet.

[6] Peiris J.S., Guan Y., Yuen K.Y. (2004). Severe acute respiratory syndrome. Nat. Med..

[7] Zaki A.M., van Boheemen S., Bestebroer T.M., Osterhaus A.D., Fouchier R.A. (2012). Isolation of a novel coronavirus from a man with pneumonia in Saudi Arabia. N. Engl. J. Med..

[8] Lee J., Chowell G., Jung E. (2016). A dynamic compartmental model for the Middle East respiratory syndrome outbreak in the Republic of Korea: A retrospective analysis on control interventions and superspreading events. J. Theor. Biol..

[9] Lee J.Y., Kim Y.J., Chung E.H., Kim D.W., Jeong I., Kim Y., Yun M.R., Kim S.S., Kim G., Joh J.S. (2017). The clinical and virological features of the first imported case causing MERS-CoV outbreak in South Korea, 2015. BMC Infect Dis..

[10] Cheng V.C.C., Wong S.C., To K.K.W., Ho P.L., Yuen K.Y. (2020). Preparedness and proactive infection control measures against the emerging Wuhan coronavirus pneumonia in China. J. Hosp. Infect..

[11] Zhu N., Zhang D., Wang W., Li X., Yang B., Song J., Zhao X., Huang B., Shi W., Lu R. (2020). A Novel Coronavirus from Patients with Pneumonia in China, 2019. N. Engl. J. Med..

[12] Huang C., Wang Y., Li X., Ren L., Zhao J., Hu Y., Zhang L., Fan G., Xu J., Gu X. (2020). Clinical features of patients infected with 2019 novel coronavirus in Wuhan, China. Lancet.

[13] WHO (2003). Summary of Probable SARS Cases with Onset of Illness from 1 November 2002 to 31 July 2003. http://www.who.int/csr/sars/country/table2004_04_21/en/index.html.

[14] WHO (2019). MERS Monthly Report November 2019.

[15] Biscayart C., Angeleri P., Lloveras S., Chaves T.D., Schlagenhauf P., Rodríguez-Morales A.J. (2020). The next big threat to global health? 2019 novel coronavirus (2019-nCoV): What advice can we give to travellers?—Interim recommendations January 2020, from the Latin-American society for Travel Medicine (SLAMVI). Travel Med. Infect. Dis..

[16] Xinhua (2020). China’s CDC Detects a Large Number of New Coronaviruses in the South China Seafood Market in Wuhan. http://www.xinhuanet.com/2020-01/27/c_1125504355.htm.

[17] Cohen J. (2020). Wuhan seafood market may not be source of novel virus spreading globally. Science.

[18] Li Q., Guan X., Wu P., Wang X., Zhou L., Tong Y., Ren R., Leung K.S.M., Lau E.H.Y., Wong J.Y. (2020). Early Transmission Dynamics in Wuhan, China, of Novel Coronavirus-Infected Pneumonia. N. Engl. J. Med..

[19] Zhou P., Yang X.-L., Wang X.-G., Hu B., Zhang L., Zhang W., Si H.-R., Zhu Y., Li B., Huang C.-L. (2020). Discovery of a novel coronavirus associated with the recent pneumonia outbreak in humans and its potential bat origin. bioRxiv.

[20] Guo Q., Li M., Wang C., Wang P., Fang Z., Tan J., Wu S., Xiao Y. (2020). Host and infectivity prediction of Wuhan 2019 novel coronavirus using deep learning algorithm. bioRxiv.

[21] Kan B., Wang M., Jing H., Xu H., Jiang X., Yan M., Liang W., Zheng H., Wan K., Liu Q. (2005). Molecular evolution analysis and geographic investigation of severe acute respiratory syndrome coronavirus-like virus in palm civets at an animal market and on farms. J. Virol..

[22] Sabir J.S., Lam T.T., Ahmed M.M., Li L., Shen Y., Abo-Aba S.E., Qureshi M.I., Abu-Zeid M., Zhang Y., Khiyami M.A. (2016). Co-circulation of three camel coronavirus species and recombination of MERS-CoVs in Saudi Arabia. Science.

[23] Cyranoski D. (2020). Mystery deepens over animal source of coronavirus. Nature.

[24] Lam T.T., Jia N., Zhang Y.W., Shum M.H., Jiang J.F., Zhu H.C., Tong Y.G., Shi Y.X., Ni X.B., Liao Y.S. (2020). Identifying SARS-CoV-2-related coronaviruses in Malayan pangolins. Nature.

[25] Ji W., Wang W., Zhao X., Zai J., Li X. (2020). Homologous recombination within the spike glycoprotein of the newly identified coronavirus may boost cross-species transmission from snake to human. J. Med. Virol..

[26] Frutos R., Serra-Cobo J., Chen T., Devaux C.A. (2020). COVID-19: Time to exonerate the pangolin from the transmission of SARS-CoV-2 to humans. Infect. Genet. Evol..

[27] Perlman S. (2020). Another Decade, Another Coronavirus. N. Engl. J. Med..

[28] WHO (2020). Novel Coronavirus (2019-nCoV) Situation Report.

[29] Wu J.T., Leung K., Leung G.M. (2020). Nowcasting and forecasting the potential domestic and international spread of the 2019-nCoV outbreak originating in Wuhan, China: A modelling study. Lancet.

[30] WHO (2003). Consensus Document on the Epidemiology of Severe Acute Respiratory Syndrome (SARS).

[31] WHO (2018). WHO MERS Global Summary and Assessment of Risk.

[32] Liu Y., Qu H.-Q., Qu J., Tian L., Hakonarson H. (2020). Expression Pattern of the SARS-CoV-2 Entry Genes ACE2 and TMPRSS2 in the Respiratory Tract. Viruses.

[33] Yu H.-Q., Sun B.-Q., Fang Z.-F., Zhao J.-C., Liu X.-Y., Li Y.-M., Sun X.-Z., Liang H.-F., Zhong B., Huang Z.-F. (2020). Distinct features of SARS-CoV-2-specific IgA response in COVID-19 patients. Eur. Respir. J..

[34] Bai Y., Yao L., Wei T., Tian F., Jin D.-Y., Chen L., Wang M. (2020). Presumed asymptomatic carrier transmission of COVID-19. JAMA.

[35] Mizumoto K., Kagaya K., Zarebski A., Chowell G. (2020). Estimating the asymptomatic proportion of coronavirus disease 2019 (COVID-19) cases on board the Diamond Princess cruise ship, Yokohama, Japan, 2020. Eurosurveillance.

[36] Zhou P., Yang X.-L., Wang X.-G., Hu B., Zhang L., Zhang W., Si H.-R., Zhu Y., Li B., Huang C.-L. (2020). A pneumonia outbreak associated with a new coronavirus of probable bat origin. Nature.

[37] Qin J., You C., Lin Q., Hu T., Yu S., Zhou X.-H. (2020). Estimation of incubation period distribution of COVID-19 using disease onset forward time: A novel cross-sectional and forward follow-up study. medRxiv.

[38] Rothe C., Schunk M., Sothmann P., Bretzel G., Froeschl G., Wallrauch C., Zimmer T., Thiel V., Janke C., Guggemos W. (2020). Transmission of 2019-nCoV Infection from an Asymptomatic Contact in Germany. N. Engl. J. Med..

[39] Kupferschmidt K. (2020). Study claiming new coronavirus can be transmitted by people without symptoms was flawed. Science.

[40] (2020). About the Present Situation of New Type Coronavirus Infectious Disease and Correspondence of Ministry of Health, Labor and Welfare (February 7 Version). https://www.mhlw.go.jp/stf/newpage_09396.html.

[41] (2020). The Second Batch of Wuhan Evacuees All Tested Negative and One Was Confirmed Infected in the First Batch. http://world.kbs.co.kr/service/news_view.htm?lang=c&Seq_Code=66621.

[42] (2020). Routine New Headquarters of the Central Accident Management Division of New Coronavirus Infection 2020-02-01. https://www.mohw.go.kr/eng/nw/nw0101vw.jsp?PAR_MENU_ID=1007&MENU_ID=100701&page=1&CONT_SEQ=352718.

[43] (2020). New Outbreaks of Corona Virus in Korea. http://www.mohw.go.kr/react/al/sal0301vw.jsp?PAR_MENU_ID=04&MENU_ID=0403&page=3&CONT_SEQ=352645.

[44] Waldrop T., Yan H. (2020). The 1st Group of Americans Evacuated from Wuhan and Quarantined over Coronavirus just Got Released. https://edition.cnn.com/2020/02/10/us/coronavirus-american-evacuees-release/index.html.

[45] (2020). Coronavirus: German Evacuation Flight from China Carried Two Infected People. DW.

[46] AFP (2020). Coronavirus: Countries Evacuate Citizens from China. https://www.nst.com.my/world/world/2020/01/561086/coronavirus-countries-evacuate-citizens-china.

[47] Wei T.T. (2020). Some Singaporeans with Symptoms of Virus Staying behind in Wuhan even as 92 Are Evacuated. The Straits Times.

[48] Yong C. (2020). Coronavirus: 2 New Cases in S’pore, Including Certis Officer Who Had Served Quarantine Orders on 2 Who Tested Positive. The Straits Times.

[49] Seckin B. (2020). Italy Reports Third Confirmed Case of Coronavirus. AA.

[50] Network C.N. (2020). Health Committee Responds to 5 Million People Leaving Wuhan: Our Common Enemy Is Disease, not Wuhan. Sina News.

[51] Farzanegan M.R., Feizi M., Gholipour H.F. (2020). Globalization and Outbreak of COVID-19: An Empirical Analysis.

[52] Chan J.F., Yuan S., Kok K.H., To K.K., Chu H., Yang J., Xing F., Liu J., Yip C.C., Poon R.W. (2020). A familial cluster of pneumonia associated with the 2019 novel coronavirus indicating person-to-person transmission: A study of a family cluster. Lancet.

[53] Iuliano A.D., Roguski K.M., Chang H.H., Muscatello D.J., Palekar R., Tempia S., Cohen C., Gran J.M., Schanzer D., Cowling B.J. (2018). Estimates of global seasonal influenza-associated respiratory mortality: A modelling study. Lancet.

[54] Taubenberger J.K., Morens D.M. (2006). 1918 Influenza: The mother of all pandemics. Emerg. Infect. Dis..

[55] Biggerstaff M., Cauchemez S., Reed C., Gambhir M., Finelli L. (2014). Estimates of the reproduction number for seasonal, pandemic, and zoonotic influenza: A systematic review of the literature. BMC Infect. Dis..

[56] Harper S.A., Fukuda K., Uyeki T.M., Cox N.J., Bridges C.B. (2005). Prevention and Control of Influenza Recommendations of the Advisory Committee on Immunization Practices (ACIP). Morb. Mortal. Wkly. Rep. Recomm. Rep..

[57] Pan H., Peto R., Abdool Karim Q., Alejandria M., Henao Restrepo A.M., Hernandez Garcia C., Kieny M.P., Malekzadeh R., Murthy S., Preziosi M.-P. (2020). Repurposed antiviral drugs for COVID-19; interim WHO SOLIDARITY trial results. medRxiv.

[58] Litvinova M., Liu Q.H., Kulikov E.S., Ajelli M. (2019). Reactive school closure weakens the network of social interactions and reduces the spread of influenza. Proc. Natl. Acad. Sci. USA.

[59] Moghadas S.M., Haworth-Brockman M., Isfeld-Kiely H., Kettner J. (2015). Improving public health policy through infection transmission modelling: Guidelines for creating a Community of Practice. Can. J. Infect. Dis. Med. Microbiol..

[60] Bootsma M.C., Ferguson N.M. (2007). The effect of public health measures on the 1918 influenza pandemic in U.S. cities. Proc. Natl. Acad. Sci. USA.

[61] Gunaratnam P.J., Tobin S., Seale H., Marich A., McAnulty J. (2014). Airport arrivals screening during pandemic (H1N1) 2009 influenza in New South Wales, Australia. Med. J. Aust..

[62] Nishiura H., Kamiya K. (2011). Fever screening during the influenza (H1N1-2009) pandemic at Narita International Airport, Japan. BMC Infect. Dis..

[63] CDC (2019). CDC Seasonal Flu Vaccine Effectiveness Studies.

[64] Plotkin S., Robinson J.M., Cunningham G., Iqbal R., Larsen S. (2017). The complexity and cost of vaccine manufacturing—An overview. Vaccine.

[65] Pong W. (2020). A Dozen Vaccine Programs under Way as WHO Declares Coronavirus Public Health Emergency. https://www.biocentury.com/article/304328/a-dozen-vaccine-programs-under-way-as-who-declares-coronavirus-public-health-emergency.

[66] Corum J., Wee S., Zimmer C. (2020). Coronavirus Vaccine Tracker. https://www.nytimes.com/interactive/2020/science/coronavirus-vaccine-tracker.html.

[67] Dong Y., Dai T., Wei Y., Zhang L., Zheng M., Zhou F. (2020). A systematic review of SARS-CoV-2 vaccine candidates. Signal Transduct. Target Ther..

[68] Feng E., Cheng A. (2020). In Quarantined Wuhan, Hospital Beds For Coronavirus Patients Are Scarce. NPR.

[69] Pickrell R. (2020). Wuhan is scrambling to fill 11 sports centres, exhibition halls, and other local venues with over 10,000 beds to create makeshift coronavirus hospitals. Bus. Insider.

[70] DXY (2020). Epidemic Map. https://3g.dxy.cn/newh5/view/pneumonia?from=timeline&isappinstalled=0.

[71] Kwok K.O., Lai F., Wei W.I., Wong S.Y.S., Tang J.W. (2020). Herd immunity–estimating the level required to halt the COVID-19 epidemics in affected countries. J. Infect..

[72] Korber B., Fischer W., Gnanakaran S., Yoon H., Theiler J., Abfalterer W., Foley B., Giorgi E., Bhattacharya T., Parker M. (2020). Spike mutation pipeline reveals the emergence of a more transmissible form of SARS-CoV-2. bioRxiv.

[73] Vergano D. (2020). Don’t Worry About The Coronavirus. Worry About The Flu. Buzzfeed News.

[74] The Lancet (2020). Emerging understandings of 2019-nCoV. Lancet.

[75] Ioannidis J. (2020). The infection fatality rate of COVID-19 inferred from seroprevalence data. medRxiv.

